# Distinct subsets of Eve-positive pericardial cells stabilise cardiac outflow and contribute to Hox gene-triggered heart morphogenesis in *Drosophila*

**DOI:** 10.1242/dev.158717

**Published:** 2018-01-15

**Authors:** Monika Zmojdzian, Svetlana de Joussineau, Jean Philippe Da Ponte, Krzysztof Jagla

**Affiliations:** GReD - INSERM U1103, CNRS UMR6293, University of Clermont Auvergne, 63000 Clermont-Ferrand, France

**Keywords:** *Drosophila*, Hox genes, Heart, Pericardial cell

## Abstract

The *Drosophila* heart, composed of discrete subsets of cardioblasts and pericardial cells, undergoes Hox-triggered anterior-posterior morphogenesis, leading to a functional subdivision into heart proper and aorta, with its most anterior part forming a funnel-shaped cardiac outflow. Cardioblasts differentiate into Tin-positive ‘working myocytes’ and Svp-expressing ostial cells. However, developmental fates and functions of heart-associated pericardial cells remain elusive. Here, we show that the pericardial cells that express the transcription factor Even Skipped adopt distinct fates along the anterior-posterior axis. Among them, the most anterior Antp-Ubx-AbdA*-*negative cells form a novel cardiac outflow component we call the outflow hanging structure, whereas the Antp-expressing cells differentiate into wing heart precursors. Interestingly, *Hox* gene expression in the Even Skipped-positive cells not only underlies their antero-posterior diversification, but also influences heart morphogenesis in a non-cell-autonomous way. In brief, we identify a new cardiac outflow component derived from a subset of Even Skipped-expressing cells that stabilises the anterior heart tip, and demonstrate non-cell-autonomous effects of Hox gene expression in the Even Skipped-positive cells on heart morphogenesis.

## Abstract (precis)

**Summary:** A new cardiac outflow component, the outflow hanging structure, is identified that originates from an anteriorly located subpopulation of Eve-expressing cells and stabilises spatial positioning of the cardiac outflow in late-stage *Drosophila* embryos.

## INTRODUCTION

The multichamber vertebrate heart is composed of contractile myocytes and a large population of nonmuscle cells that are required for cardiac morphogenesis and function. The epicardium, which is composed of nonmuscle cells, forms an epithelial layer on the surface of the myocardium (Brade et al., 2013), and is known to be a source of signals that influence myocyte proliferation, myocardium growth and maturation (Schlueter and Brand, 2012).

Unlike in vertebrates, in *Drosophila* the heart organ remains a linear tube. However, it also comprises contractile cardiomyocytes and a population of nonmuscle cells, including the heart-associated pericardial cells (Cripps and Olson, 2002; Bodmer and Frasch, 2010). It is organised into segmental units with specific expression of cardiac identity genes, which guide the diversification of cardioblasts and pericardial cells. Morphologically, the cardiac tube can be divided into a wider posterior part called the heart proper, with functional ostia making an inflow tract (Gajewski et al., 2000; Molina and Cripps, 2001), and a narrow anterior part called the aorta, with a funnel-shaped outflow at its anterior extremity (Zikova et al., 2003; Zmojdzian et al., 2008). The anterior-posterior (A-P) patterning of the heart tube is controlled by Hox genes, which are expressed along the A-P axis (Lo et al., 2002; Lovato et al., 2002; Ponzielli et al., 2002; Lo and Frasch, 2003; Monier et al., 2007).

The pericardial cells can be divided into Tinman (Tin)-, Even Skipped (Eve)- and Odd Skipped (Odd)-expressing cells, and a subpopulation of cells expressing the Iroquois complex (Ward and Skeath, 2000; Alvarez et al., 2003; Mirzoyan and Pandur, 2013). Among them, Tin and Eve counterparts Nkx2.5 and Evx2, respectively, are expressed in the vertebrate heart (Harvey, 1996; Fujioka et al., 2005). Pericardial cells of the same class can also adopt distinct fates depending on their A-P locations. For example, the Odd-expressing cells from the thoracic segments differentiate into the lymph glands, which are hematopoietic organs (Evans et al., 2003), whereas the Odd-positive cells from the abdominal segments give rise to the pericardial nephrocytes (Ward and Skeath, 2000). This A-P subdivision of Odd-positive cells appears to be under the control of Hox genes, given that the formation of the lymph glands requires Antennapedia and Bithorax complex gene functions (Mandal et al., 2004; Perrin et al., 2004).

Here, we report that the Eve-positive pericardial cells (EPCs) also adopt distinct fates according to their A-P position. It has been previously shown that EPCs originating from the thoracic segments give rise to the wing hearts (WHs), adult fly organs that are essential for wing maturation and flight ability (Tögel et al., 2008). Here, we identify an additional anteriorly located subpopulation of EPCs that differentiate into a specialised cardiac outflow-associated structure we call the outflow hanging structure (OHS). Our data indicate that the OHS stabilises spatial positioning of the cardiac outflow in late-stage embryos, and demonstrate that Hox genes control the A-P diversification of EPCs.

## RESULTS AND DISCUSSION

### Diversity of Eve-positive pericardial cells: the most anterior EPCs form the outflow hanging structure

**Fig. 1.**
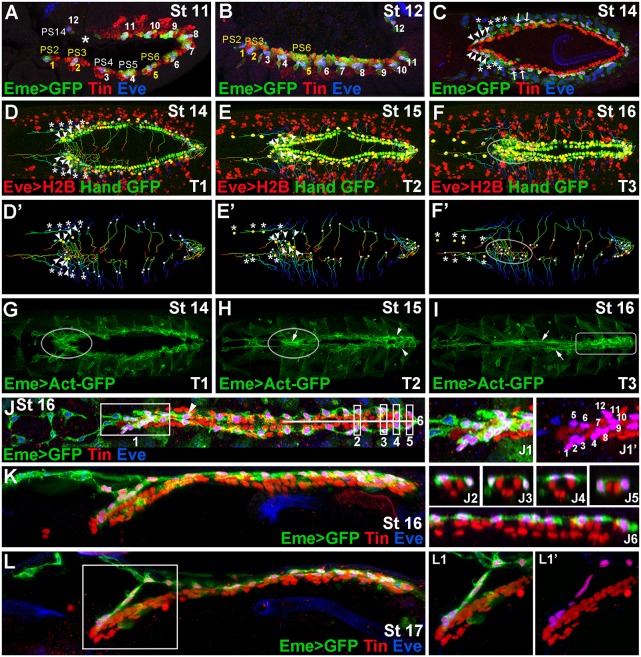
**Diversity of EPCs and formation of the OHS.** (A,B) Lateral views from Eme>mcd8GFP stage 11 (A) and 12 (B) embryos showing 12 EPC clusters in dorsal mesoderm. The parasegments (PSs) from which OHS EPCs originate are coloured yellow. Asterisk in A indicates missing EPCs in PS13. (C) Dorsal view of a stage 14 embryo. Arrowheads indicate the OHS EPCs from PS2 and PS3; asterisks indicate detaching WH EPCs. The OHS EPCs from PS6 are indicated by arrows. (D-F′) Dorsal views of stage 14-16 living Hand-nlsGFP;Eme>H2B embryos showing cell tracking (lines) of migrating EPCs (white or yellow dots). (G-I) Dorsal views of Eme>lifeAct-GFP stage 14-16 living embryos. Leading edge activity of the migrating OHS EPCs is increased (G, circled). As the heart closure proceeds (H) the OHS EPCs become aligned (circled) with the apparent actin cable (arrow), whereas the heart proper EPCs (arrowheads) form a network of interconnected cells. At stage 16 (I), the actin cables (arrows) link the aorta EPCs, whereas the heart proper-associated EPCs (rectangle) display a patched actin enrichment. (J) Dorsal view of a stage 16 Eme>mcd8GFP embryo. (J1,J1ʹ) The OHS EPCs appear highly compacted and located dorsally above the heart tip. The EPCs associated with the aorta, except the first pair (arrowhead), lie more laterally and are interspaced according to the segmental register. The heart proper EPCs are located above or lateral to the cardioblasts (J2-J6), and appear as a dorsally connected network of cells. (K,L) Lateral views of stage 16 (K) and 17 (L) embryos stained as above and showing the arrangement of the heart-associated EPCs, which are interconnected. The OHS EPCs (L1,L1′) make a link between the bent anterior aorta and the epidermis.

The heart-associated EPCs are a subpopulation of pericardial cells of well-defined origin (Park et al., 1998; Carmena et al., 1998a,b, 2002). They can be detected from embryonic stage 11 (Fig. 1A) in dorsally located clusters of cells also harbouring the precursors of DA1 muscle, except for the most posterior cluster (PS14), which harbours only one EPC (Fig. 1B). As previously reported (Tögel et al., 2008) the EPCs from the PS4 and PS5 (asterisks in Fig. 1C-F′) detach from the cardiac primordium, and differentiate into the WHs. Here, we observe that EPCs from the PS2 and PS3 (arrowheads, Fig. 1C-E′), and also those from PS6 (arrows, Fig. 1C), display distinct behaviour; in late-stage embryos they form a cluster of cells lying on the dorsal side of the heart tip (Fig. 1J-L). As revealed by cell-tracking experiments (Fig. 1D-F′, Movies 1 and 2) the PS2- and PS3-derived EPCs underwent individual posteriorly oriented migration towards the dorsal midline. In parallel, EPCs from the PS6 migrated dorsally with slightly anterior orientation (Fig. 1D-F′, Movies 1 and 2), connecting to each other and filling the gap after anterior migration of WH precursors from PS4 and PS5. In addition, as revealed by live *in vivo* Actin imaging (Fig. 1G-I, Movie 3), the heart tip-associated EPCs appeared rich in actin (outlined area in Fig. 1G,H), and formed an axial structure with an apparent actin cable (arrow, Fig. 1H; Movie 3). By contrast, EPCs linked to posterior aorta remained in bilateral rows with lower actin levels, and only in late-stage embryos did they form actin cables underlying cellular connections (arrows, Fig. 1I, Movie 3). Moreover, the EPCs associated with the heart proper displayed network-like cellular arrangements with high actin accumulation (Fig. 1H,I; Movie 3).

Thus, our data indicate that EPCs diversify within the A-P axis into four cell subsets. The PS2, PS3 and PS6-derived EPCs are compacted, and form an axial structure (‘1’ in Fig. 1J) on the dorsal side of the heart tip; the PS4 and PS5 EPCs give rise to the WHs; the posterior aorta EPCs stand as bilateral rows of pericardial cells (Fig. 1J); and the heart proper EPCs lie at dorsal or dorso-lateral positions on both sides of Tin-expressing cardioblasts (Fig. 1J^2-6^), forming an epicardium-like structure. Interestingly, except for the WH precursors, which migrate away from the heart field, all the other EPCs, irrespective of their individual properties, form a network that is interconnected via cellular protrusions.

Regarding the first EPC subset, the most anterior PS2-EPCs aligned from the dorsal side with a pair of cardioblasts to which are attached the cardiac outflow muscles (COMs) and the heart-anchoring cells (HANCs) (Zikova et al., 2003). This suggests that these EPCs could be a new component of the cardiac outflow (Zikova et al., 2003; Zmojdzian et al., 2008), a possibility supported by the observation that, in late-stage embryos, the anterior EPCs differentiate into a structure that connects cardiac outflow to the dorsal epidermis (Fig. 1L,L^1^,L^1′^, Movie 4). Based on all these observations, we call the cluster of EPCs derived from PS2, PS3 and PS6 the outflow hanging structure (OHS).

### Regional Hox gene expression in EPCs

**Fig. 2.**
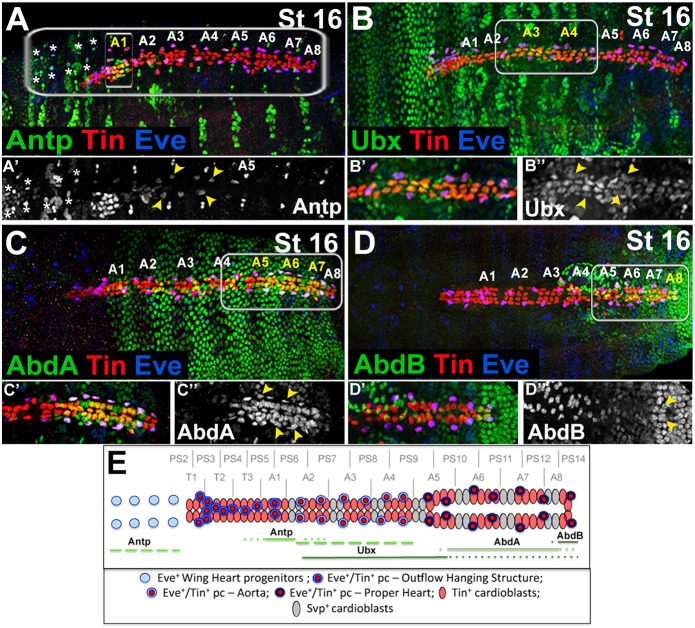
**Expression pattern of Hox proteins in the EPCs.** (A-D″) Dorsal views of stage 16 embryos showing expression of Hox proteins. (A′-D″) Higher magnification views corresponding to areas framed by rectangles in A-D. (A,A′) Antp expression in the cardiac cells is confined to the A1 segment, including the A1 EPCs. There is also prominent expression of Antp in the WH EPCs (asterisks) and in EPCs from A2-A4 (yellow arrowheads). (B-B″) Ubx labels cardioblasts and pericardial cells, including EPCs (yellow arrowheads) from A3 and A4, and also cardiac cells from A2 and A5. Faint Ubx expression is seen in the heart proper. (C) High AbdA expression is detected in the cardioblasts and pericardial cells, including the EPCs (yellow arrowheads), from A6, A7 and the posterior part of A5. (D) AbdB expression is restricted to the most-posterior A8 cardioblasts and the pericardial cells, including the last pair of EPCs (yellow arrowheads). (E) Hox protein expression in the heart, including the EPCs. Segmental and parasegmental registers are depicted. Solid lines indicate the extent of Hox expression in the EPCs and cardioblasts. Dotted lines indicate the extent of Hox expression in cardioblasts. Dashed green lines indicate extended Antp expression in EPCs. No Hox expression could be detected in OHS EPCs from PS2 and PS3.

Anterior-posterior diversification of EPCs suggests involvement of the Hox genes, which determine the A-P polarity of the embryonic fly heart (Lovato et al., 2002; Lo et al., 2002; Ponzielli et al., 2002). Previous analyses of the homeotic gene expression revealed that *Antennapedia* (*Antp*), *Ultrabithorax* (*Ubx*), *Abdominal A* (*AbdA*) and *Abdominal B* (*AbdB*) displayed regional expression in cardiac cells (Lovato et al., 2002; Lo et al., 2002; Ponzielli et al., 2002). Among them, *Antp*, *Ubx* and *AbdA* are expressed not only in the cardioblasts, but also in the pericardial cells (Lo et al., 2002). However, the identity of Hox-expressing pericardial cells has not been determined. We therefore tested whether expression of Antp, Ubx, AbdA and AbdB could be detected in EPCs, and found that all the EPCs, except the eight that are most anterior, expressed one of the four Hox genes tested (Fig. 2). Antp, besides its predominant expression in the cardiac and pericardial cells from the A1, also displays strong expression in WH EPCs (Fig. 2A,A′,E), and was also detected in the EPCs within the A2 to A4 segments (Fig. 2A′,E). Similarly, Ubx, though at lower levels, was expressed in EPCs within abdominal segments A3 and A4 (Fig. 2B-B″,E), and high AbdA expression was found in EPCs from A5, A6 and A7 (Fig. 2C-C″,E). Finally, AbdB expressed in the most-posterior A8 segment within the cardiac domain was also detected in the last pair of EPCs (Fig. 2D-E). These observations support the possibility that the Hox genes control the anterior-posterior diversification of EPCs as they do for the cardioblasts (Lo et al., 2002). The fact that we did not detect any Hox gene expression in the eightmost anterior EPCs (Fig. 2E) that build the OHS highlights the distinct properties of this Eve-positive cell subset.

### OHS contributes to the stabilisation of the cardiac outflow

**Fig. 3.**
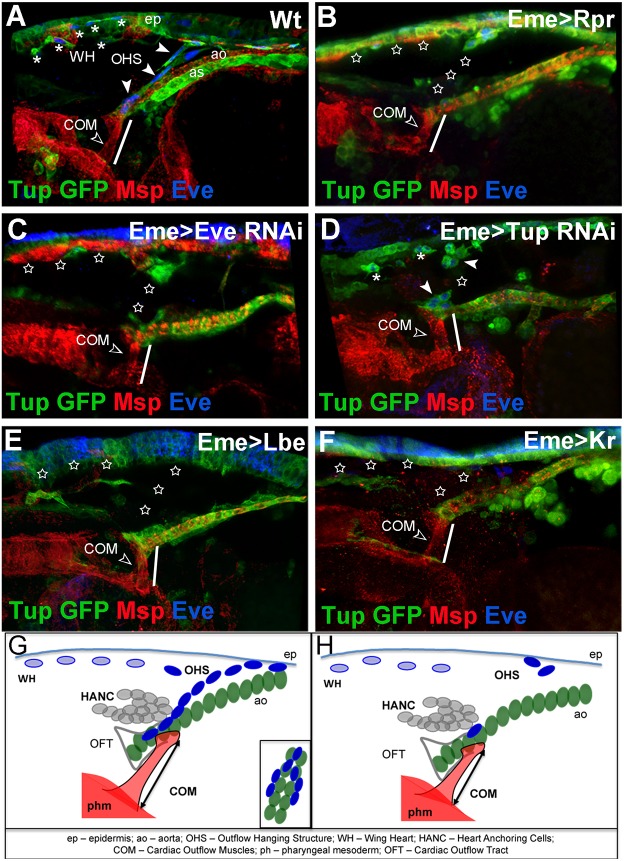
**The OHS is required for cardiac outflow positioning.** (A-F) Lateral views of the cardiac outflow region from Tup-GFP late stage 16 embryos. Tup-GFP reveals EPCs, aorta (ao), amnioserosa (as) and epidermis (ep); Msp300 stains COMs (open arrowhead) and cardiac cells; and Eve labels EPCs. (A) Wild-type embryo: the OHS EPCs are indicated by arrowheads and WH EPCs by asterisks. (B) Eme>Rpr embryo in which EPCs were ablated (stars). The COMs appear shorter (white line) than in wild type (compare A with B). (C) EPC-specific *eve*RNAi results in partial loss of OHS and WH EPCs (stars), and affects COMs length (white line). (D) EPC-targeted *tup*RNAi results in partial loss of EPCs. The COMs appear shorter (white line). (E,F) The *eme*-driven misexpression of Lbe (E) or Kr (F) represses *eve*, leading to affected specification of OHS and WH EPCs, and shortening of COMs (white lines). (G) Cellular components of cardiac outflow, including OHS. The OHS EPCs (dark blue) connect the tip of the anterior aorta (green) to the epidermis and, like COMs, are attached on both sides of the cardiac outflow. (H) Affected positioning of the cardiac outflow associated with COM shortening in the contexts where the OHS is lost.

To understand the developmental function of the OHS, we performed genetic ablation, RNAi knock-down and misexpression experiments using eme-GAL4, which drives expression in all EPCs, including the OHS (Fujioka et al., 2005). We found that the Tailup (Tup)-GFP line, with its strong expression in the heart tube and in the OHS, offered a perfect tool with which to follow cardiac outflow phenotypes, including spatial positioning (Fig. 3A, Movie 5). We first observed that in late-stage 16 embryos, the elimination of the OHS by inducing apoptosis in EPCs led to smaller cardiac outflow muscles (COMs) attached to the cardiac outflow from the ventral side (Fig. 3B, Table 1). This suggests that the OHS might counteract tension generated by the COMs when they start to contract. Consequently, ablation of the OHS causes a ventral shift of the heart tip and COM shortening (Fig. 3B), but does not have any impact on outflow shape or on HANC cells (Fig. S1). We also found that Eve and Tup both played instructive roles in OHS specification, because in *EveRNAi* (Fig. 3C) and *TupRNAi* (Fig. 3D) contexts, the OHS was absent or severely affected. OHS defects observed in the Tup knockdown context are consistent with its important role in the proliferation and differentiation of cardiac cells (Mann et al., 2009; Pandur et al., 2013). OHS loss was also observed in embryos expressing the identity genes *lbe* or *Kr* in EPCs (Fig. 3E,F). Both these cell fate-specifying genes act as potent repressors of Eve (Jagla et al., 2002; Carmena et al., 1998a), further highlighting the pivotal role of Eve in the development of the OHS. As in all cases analysed, OHS loss was associated with shortening of COMs (Table 1), and consequently a ventral shift of the heart tip, we conclude that the OHS could play a stabilising role for the cardiac outflow. We hypothesise that the OHS, attached from the dorsal side to the same pair of cardioblasts to which the COMs are attached ventrally, counteracts ventral pooling of the cardiac outflow by the COMs, thus stabilising the cardiac outflow position (Fig. 3G,H). Tight connections and compaction of the EPCs that build the OHS compared with other EPCs allow us to speculate that the OHS develops into a specialised cluster of cells that is able to balance the force generated by the COMs in late-stage embryos and thus ensure a proper cardiac outflow position.

**Table 1.**
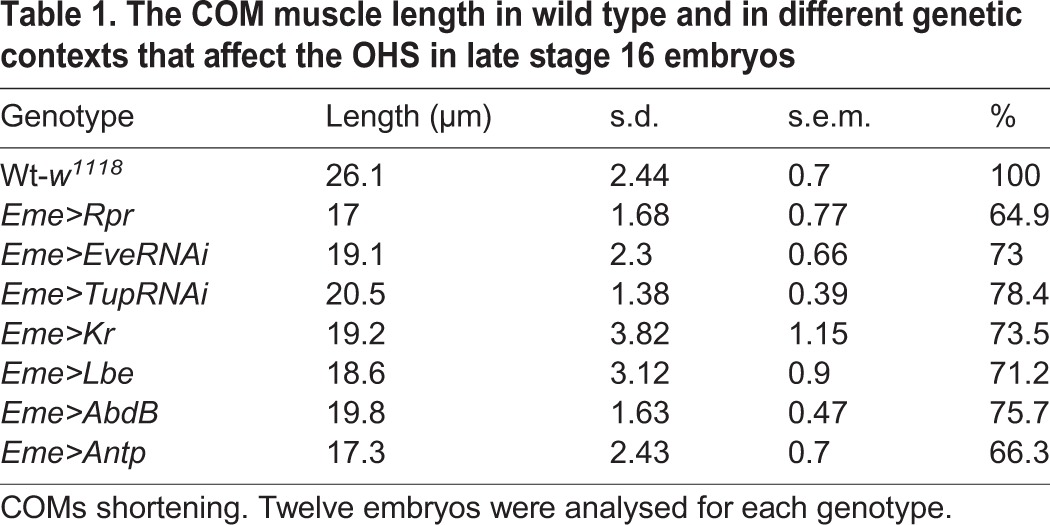
**The COM muscle length in wild type and in different genetic contexts that affect the OHS in late stage 16 embryos**

### Hox genes trigger anterior-posterior diversification of EPCs

**Fig. 4.**
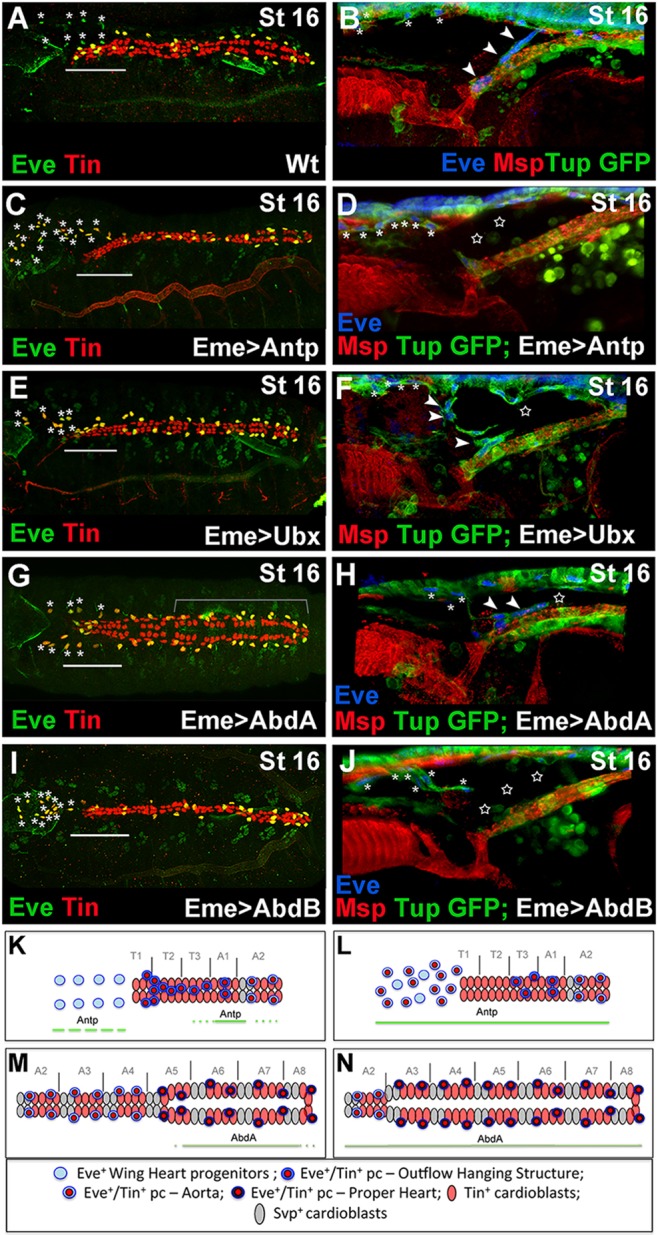
**Hox genes control EPCs diversity.** (A,C,E,G,I) Dorsal views of late stage 16 embryos stained with Tin and Eve. (B,D,F,H,J) Lateral views of the cardiac outflow region from late stage 16 Tup-GFP embryos stained for GFP, Eve and Msp300. (A,B) In the wild-type embryos, the OHS EPCs are associated with the anterior aorta (A, above the line; B, arrowheads) and the WH EPCs are located anteriorly to the heart (asterisks). (C,D) In the Eme>Antp context, an increased number of EPCs adopt the WH fate (C,D, asterisks) and the OHS EPCs are almost absent (C, above the line; D, stars). (E,F) Eme-targeted overexpression of Ubx disrupts OHS morphogenesis (F, star) without shifting the identity of OHS EPCs to WH fate (E, above the line). Few OHS EPCs (arrowheads) are located more anteriorly compared with wild type (A). Asterisks indicate WH cells. The heart proper appears thinner. (G,H) In eme>AbdA embryos, the WH EPCs (asterisks) migrate more slowly, the string of OHS EPCs is broken (F, star) and the aorta is enlarged (G, bracket). (I,J) The Eme-driven expression of AbdB leads to a reduced EPC number. The OHS is missing (J, stars) and a few persisting EPCs adopt the WH fate (I, asterisks). In all EPC-targeted Hox misexpression experiments, the specification of the OHS EPCs is affected and the WH EPCs do not lose Tin expression. (K) Anterior aorta and WH progenitors in wild-type context. Solid and broken lines indicate Antp expression. (L) The shift in EPC identity to WH fate in the Eme-targeted Antp overexpression context. (M) The heart proper and posterior aorta organization with marked AbdA expression (solid line). (N) The extended heart proper phenotype observed in embryos with Eme-driven AbdA overexpression.

To understand the role of Hox genes in EPCs, we tested the effects of their extended expression using the eme-Gal4 driver (Fig. 4). Intriguingly, generalised to all EPCs, expression of Antp led to an increased number of EPCs that adopted a WH-like fate (Fig. 4C, Table S1). In contrast to wild-type WH precursors (Fig. 4A), those with forced Antp did not lose Tin expression (Fig. 4C). As generalised Antp expression also resulted in a complete loss of OHS-forming EPCs (Fig. 4D, stars) we might expect Antp to drive a shift in EPC identity to WH cells (Fig. 4K,L). This possibility is supported by loss of migrating WH progenitors and accumulation of EPCs at the OHS location observed in Eme>AntpRNAi and in *Antp* mutant contexts (Fig. S2, Table S1). By contrast, generalised Antp expression (Fig. 4C) or loss of function (Fig. S2) did not seem to modify EPCs associated with the aorta and the heart proper (Fig. 4C). We also observed that ectopic expression of Ubx did not affect the number of WH cells or their migration (Fig. 4E), but the positioning of OHS EPCs was affected, so that they were unable to build the OHS correctly (Fig. 4E,F). We also noted a reduced diameter of the heart proper in about 23% of Eme>Ubx embryos (Fig. 4E, Table S2), suggesting that Ubx functions in EPCs non-cell-autonomously to instruct the A-P patterning of the cardiac tube. In contrast to Ubx, ectopic expression of AbdA in all EPCs led to the formation of an enlarged aorta (21% of Eme>AbdA embryos; Table S2) resembling the heart proper (Fig. 4G, bracket; Fig. 4M,N). This AbdA phenotype resembles that induced by expression of AbdA in all cardiac cells (Lo et al., 2002; Lovato et al., 2002; Ponzielli et al., 2002; Perrin et al., 2004), and indicates a non-cell-autonomous AbdA function in EPCs that is also supported by the aorta-like heart proper phenotype in Eme>AbdARNAi and in *AbdA* mutants (Fig. S2, Table S2). Importantly, OHS formation was compromised when its precursors expressed AbdA (Fig. 4H, Table S2). Finally, expressing AbdB in all EPCs also had a dramatic effect on OHS formation (Fig. 4I,J, stars), suggesting that the most anterior EPCs are set up as Hox-free cells, and that this feature is instrumental for the acquisition of their properties.

We thus provide evidence for heterogeneity of the EPCs, which adopt distinct characteristics and fulfil different developmental roles depending on their A-P positions. Within the anterior region, this Hox-triggered diversification of EPCs results in the specification of Antp-positive WH precursors and the most anterior Antp-negative EPCs, which give rise to a new component of the cardiac outflow: the outflow hanging structure.

## MATERIALS AND METHODS

### *Drosophila* stocks

*Drosophila* stocks were maintained at 25°C. The *w^1118^* strain was used as wild type. The targeted expression experiments were performed using the UAS-GAL4 system (Brand and Perrimon, 1993) on the following GAL4 and UAS lines: eme-GAL4 (kindly provided by R. Bodmer, Burnham Institute, San Diego, CA, USA); UAS-Rpr (BL5824), UAS-Antp (BL7301), UAS-Ubx (BL911), UAS-AbdA (BL912), UAS-AbdB (BL913), UAS-mcd8-GFP (BL32184) and UAS-LifeAct-GFP (BL58718) from Bloomington Stock Center; UAS-Tup RNAi (45859) and UAS-Eve RNAi (9284) from VDRC; UAS-Tin (kindly provided by M. Frasch, Erlangen-Nürnberg University, Germany); UAS-Lbe (Jagla et al., 1997); and UAS-Kr (a gift from G. Vorbrüggen, Max Planck Institute, Goettingen, Germany). The following additional lines were used: UAS-Antp RNAi (101774) and UAS-AbdA RNAi (106155) from VDRC; Antp25 (3020) from Bloomington Stock Center; and AbdAM1 (kindly provided by L. Perrin; Aix-Marseille University, France). The Tup-GFP line was kindly provided by Stephan Thor (Linköping University, Sweden), the Hand-GFP line was a gift from E. Olson (Utah University, Salt Lake City, USA) and the UAS-H2B::YFP line was kindly provided by Michel Gho (IBPS, Paris, France). UAS-Ubx was re-balanced with *TM3*, *twi-lacZ,* and homozygous mutant embryos were selected by absence of β-galactosidase staining.

### Embryo staining and imaging

Staged embryos were dechorionated, fixed, blocked for 1 h at room temperature in 20% horse serum in PBT and then incubated with primary and secondary antibodies according to a standard procedures (Jagla et al., 1997). The primary antibodies used were: goat anti-GFP (1:500, Abcam, ab 5450), chicken anti-β galactosidase (1:1000, Abcam, ab 9361), rabbit anti-Tin (1:1000, kindly provided by M. Frasch), guinea-pig anti-Msp300 (1:2500, kindly provided by T. Volk; Weizmann Institute of Science, Rehovot, Israel), rat and rabbit anti-Eve (1:200, kindly provided by D. Kosman, University of California, San Diego, USA); mouse anti-Antp (8C11; 1:50), mouse anti-Ubx (FP3.38; 1:50), mouse anti-AbdB (1A2E9; 1:50) from Developmental Studies Hybridoma Bank (University of Iowa); goat anti-AbdA (1:500; dH-17, sc-27063) from Santa Cruz; and mouse anti-Lbe (1:2500; Jagla et al., 1997).

The secondary antibodies used were: donkey anti-rabbit, donkey anti-rat, donkey anti-guinea pig, donkey anti-goat, donkey anti-chicken and donkey anti-mouse (Jackson Immuno Research Laboratories) conjugated to Alexa 488, CY3 or CY5 fluorochromes (dilution 1:300); and donkey anti-rabbit conjugated to Alexa 405 fluorochrome (dilution 1:300) (Jackson Immuno Research Laboratories).

*In vivo* imaging of cardiac cells was performed from stage 14 to 16 over 2 min (Eme>LifeAct GFP) or 6 min (Eme>H2B YFP; Hand GFP) intervals. All images were acquired on a Leica SP5 or SP8 confocal microscope and analysed using Imaris software (Bitplane).

## Supplementary Material

Supplementary information
