# Supplementary material for: Distinct subsets of Eve-positive pericardial cells stabilise cardiac outflow and contribute to Hox gene-triggered heart morphogenesis in Drosophila
Source: Development. 2018 Jan 15;145(2):dev158717. doi: 10.1242/dev.158717 (PMC5825839; doi:10.1242/dev.158717)
Supplement: Supplementary information [file develop-145-158717-s1.pdf]

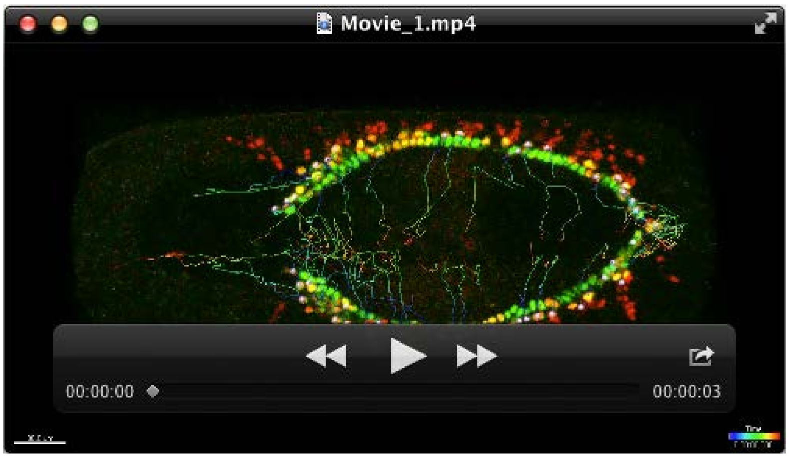

Movie 1

A time-lapse movie showing the heart closure from Hand-GFP; Eme>H2B expressing embryo. Visible are the cardioblasts (green) and EPCs (yellow) with their migratory paths (lines). The OHC EPCs stay associated with the cardioblasts and migrate toward the dorsal midline, whereas the WH EPCs detach from the heart field and move anteriorly.

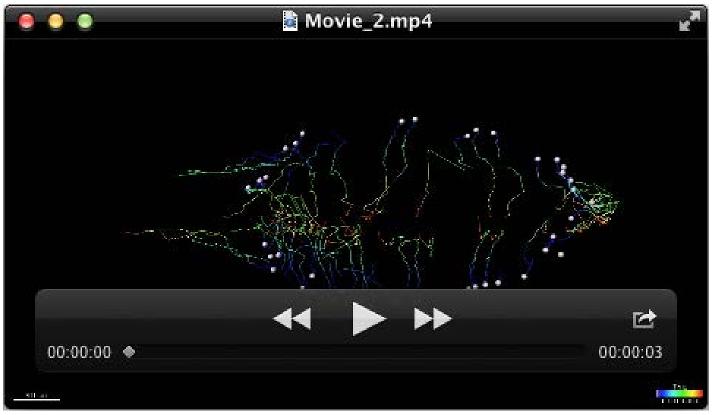

Movie 2

A time-lapse movie showing the EPCs tracking.

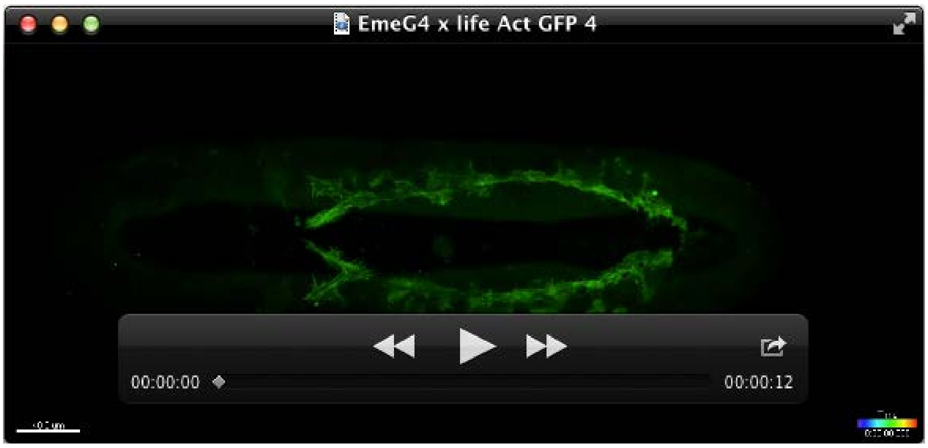

Movie 3

A time-lapse movie showing the migration and the filopodial activity of EPCs visualized in Eme>lifeAct-GFP embryo. During the heart closure EPCs appear interconnected and arranged in the bilateral rows. Notice filopodial and lamelipodial activity and the formation of an actin cable that link the compacted OHC EPCs. The WH EPCs migrate collectively and stay interconnected among them. The heart proper associated EPCs appear highly enriched in actin and form a network of cells.

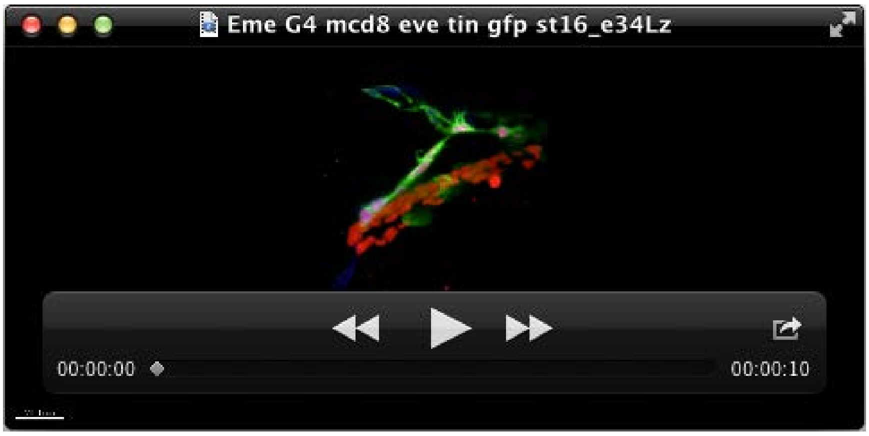

Movie 4

A 3D reconstruction of the cardiac outflow region (horizontal rotation) from Eme>mcd8GFP stage 16 embryo stained for Eve (blue) and Tin (red). Notice the arrangement and position of OHS EPCs connecting the heart tip with epidermis.

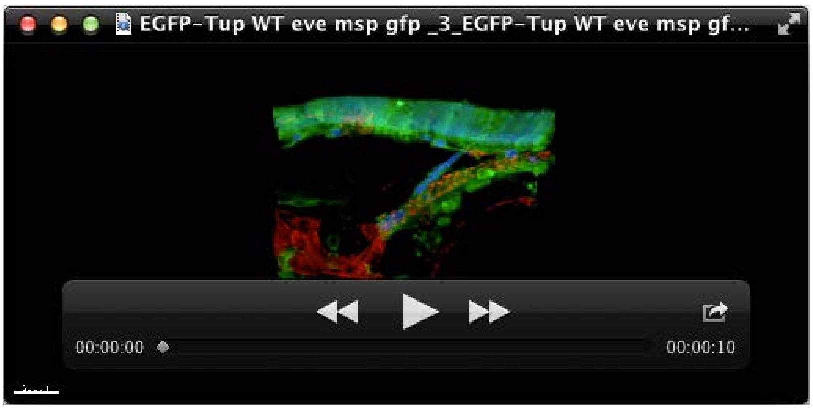

Movie 5

A 3D reconstruction of the cardiac outflow region (horizontal rotation) from Tup-GFP stage 16 embryo showing Tup expression (green) in EPCs, heart, amnioserosa and epidermis. Msp300 staining (red) reveals the COM muscle and cardiac cells. Note the position of the most anterior Eve cells (blue) above the COMs attachment to the heart and the entire OHS architecture.

Development • Supplementary information

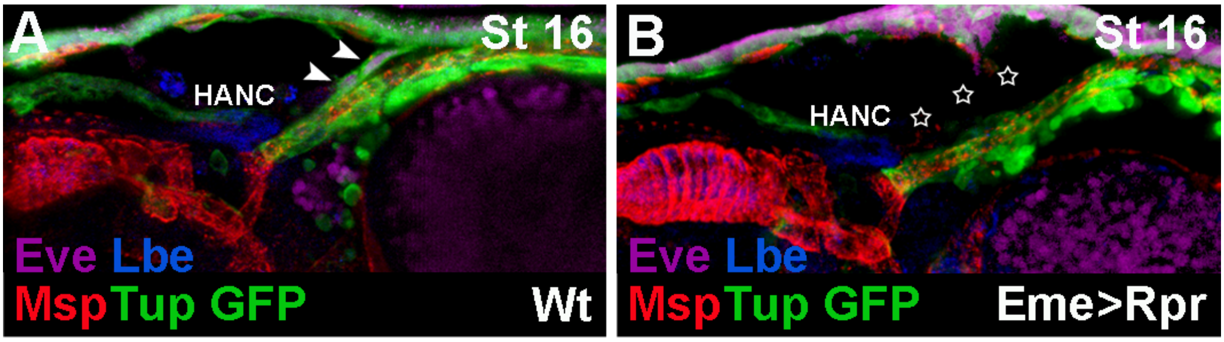

Fig. S1.

The OHS ablation has no influence on the HANC cells. (A) Lateral view of the cardiac outflow region from Tup-GFP late stage-16 embryo. The EPCs forming OHS (magenta) are indicated by arrowheads. Heart and COMs are stained with Msp300 (red) and the HANC cells with Lbe (blue). (B) A similar view of the 16-stage embryo in which the OHS was ablated by targeted induction of apoptosis using Eme-GAL4; UAS-Rpr. Notice that the OHS is absent (open arrow) and the HANCs overlapping the anterior aorta are not affected.

Development • Supplementary information

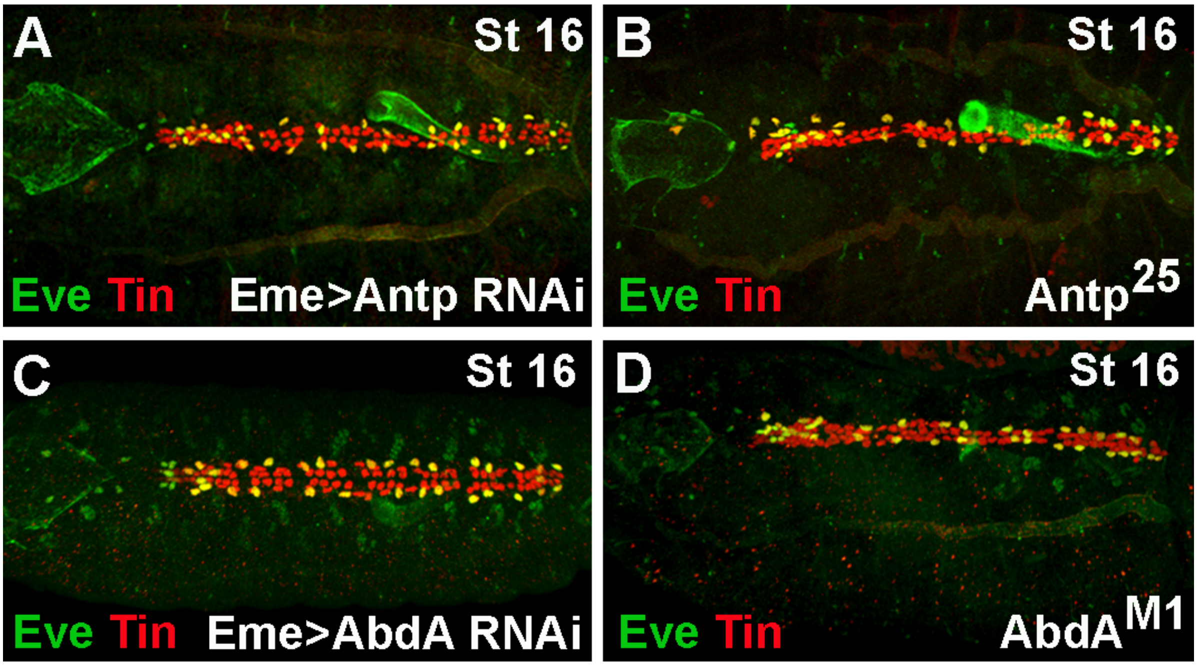

Fig. S2.

Antp and AbdA downregulation affects the WH migration and heart morphology. Dorsal views of late stage-16 embryos stained with Tin and Eve. In the Eme>AntpRNAi (A) and *Antp* mutants (B) the migration of WH progenitors is affected and accumulation of EPC at the OHS location is observed. The downregulation of AbdA in Eme>AbdARNAi context (C) and its loss in mutant context (D) led to aorta-like heart proper phenotype. For wild type view refer to Fig. 4A.

Supplementary Tables

Table S1. The WH progenitors number in embryos with deregulated Antp or AbdB expression in Eve cells.

Genotype	WH	SD	ME	%
Wt^{1118}	8	0	0	100
Eme>Antp (n=14)	14,78	2,32	0,62	184,75
Eme>AbdB (n=13)	11,23	3,7	1,02	140,37
Eme>Antp RNAi (n=11)	6,27	1,61	0,48	78,37

Table S2. The heart morphological changes in embryos with deregulated AbdA and Ubx expression in Eve cells.

Genotype	Extended proper heart (%)	Aorta-like heart (%)
Eme>AbdA (n=14)	21	-
Eme>AbdARNAi (n=14)	-	31
AbdA^{M1} (n=11)	-	91
Eme>Ubx (n=13)	-	23
